# Basic characteristics of plasma rich in growth factors (PRGF): blood cell components and biological effects

**DOI:** 10.1002/cre2.26

**Published:** 2016-03-18

**Authors:** Kazuhiko Nishiyama, Toshimitsu Okudera, Taisuke Watanabe, Kazushige Isobe, Masashi Suzuki, Hideo Masuki, Hajime Okudera, Kohya Uematsu, Koh Nakata, Tomoyuki Kawase

**Affiliations:** ^1^ Tokyo Plastic Dental Society Kita‐ku Tokyo Japan; ^2^ Division of Oral and Maxillofacial Surgery, Institute of Medicine and Dentistry Niigata University Niigata Japan; ^3^ Division of Oral Bioengineering, Institute of Medicine and Dentistry Niigata University Niigata Japan; ^4^ Bioscience Medical Research Center Niigata University Medical and Dental Hospital Niigata Japan

**Keywords:** Plasma rich in growth factors, platelet‐rich plasma, platelets, white blood cells

## Abstract

Platelet‐rich plasma (PRP) is widely used in regenerative medicine because of its high concentrations of various growth factors and platelets. However, the distribution of blood cell components has not been investigated in either PRP or other PRP derivatives. In this study, we focused on plasma rich in growth factors (PRGF), a PRP derivative, and analyzed the distributions of platelets and white blood cells (WBCs). Peripheral blood samples were collected from healthy volunteers (*N* = 14) and centrifuged to prepare PRGF and PRP. Blood cells were counted using an automated hematology analyzer. The effects of PRP and PRGF preparations on cell proliferation were determined using human periosteal cells. In the PRGF preparations, both red blood cells and WBCs were almost completely eliminated, and platelets were concentrated by 2.84‐fold, whereas in the PRP preparations, both platelets and WBCs were similarly concentrated by 8.79‐ and 5.51‐fold, respectively. Platelet counts in the PRGF preparations were positively correlated with platelet counts in the whole blood samples, while the platelet concentration rate was negatively correlated with red blood cell counts in the whole blood samples. In contrast, platelet counts and concentration rates in the PRP preparations were significantly influenced by WBC counts in whole blood samples. The PRP preparations, but not the PRGF preparations, significantly suppressed cell growth at higher doses in vitro. Therefore, these results suggest that PRGF preparations can clearly be distinguished from PRP preparations by both inclusion of WBCs and dose‐dependent stimulation of periosteal cell proliferation in vitro.

## Introduction

Platelet‐rich plasma (PRP) is a source of growth factors that promote wound healing and tissue regeneration (Marx et al. 1998) and is widely used in a variety of fields involving regenerative therapy (Kawase 2015). In addition to platelet‐derived growth factors, PRP provides fibrinogen, which is converted into insoluble fibrin fibers, to support cell adhesion and control the delivery of growth factors (Kawase et al. 2003). Furthermore, PRP provides anti‐inflammatory factors and anti‐bacterial peptides to optimize the local environment by suppressing inflammatory responses (El‐Sharkawy et al. 2007; Cieslik‐Bielecka et al. 2012b; Tohidnezhad et al. 2012; Burnouf et al. 2013). Because the augmentation of inflammatory responses delays or suppresses wound healing and tissue regeneration, it is important to control acute inflammation to induce the best performance of growth factors. Therefore, it can be speculated that when a regenerative action is combined with an anti‐inflammatory action, these actions will exert synergistic regenerative effects at sites of PRP application.

Along with platelets, high concentrations of white blood cells (WBCs) are found in PRP preparations. WBCs are known to release pro‐inflammatory cytokines, such as interleukin‐6. The presence of WBCs in PRP preparations may augment the inflammatory response at the application sites (Anitua et al. 2015b). However, in various clinical and preclinical animal studies (Burnouf et al. 2013; Mariani et al. 2014; McCarrel et al. 2014), PRP has frequently been observed to suppress infection and inflammation. These data suggest that PRP preparations possibly contain significant amounts of anti‐inflammatory cytokines and anti‐bacterial peptides (Cieslik‐Bielecka et al. 2012b; Tohidnezhad et al. 2012; Burnouf et al. 2013) that control acute inflammation. Choukroun (Cieslik‐Bielecka et al. 2012a), a developer of platelet‐rich fibrin (PRF), has claimed that appropriate numbers of WBCs should be contained in PRP and its derivatives to facilitate wound debridement. Based on his concept, Choukroun recently improved PRF (Dohan et al., 2006) to an advanced form (A‐PRF) that is further enriched with WBCs (Ghanaati et al. 2014). In contrast, based on his belief, Anitua developed PRGF by eliminating WBCs from PRP (Anitua et al. 2015a,2015b). To our knowledge, although adverse events or complications have not yet been reported for either derivative, the use of WBCs for healing and regeneration remains controversial in clinical settings.

In this study, we examined the fractionation of WBCs and platelets during PRGF preparations and compared these characteristics with those of PRP preparations. To obtain the maximum benefit from growth factors, it is usually thought that platelets should be maximally concentrated; however, if WBCs are simultaneously concentrated in the platelet fraction, the positive effects of growth factors may be reduced. To address this matter, we also examined the effects of PRGF and PRP preparations on the proliferation of human periosteal cells in vitro.

## Materials and Methods

### Preparation of plasma rich in growth factors

**Figure 1:**
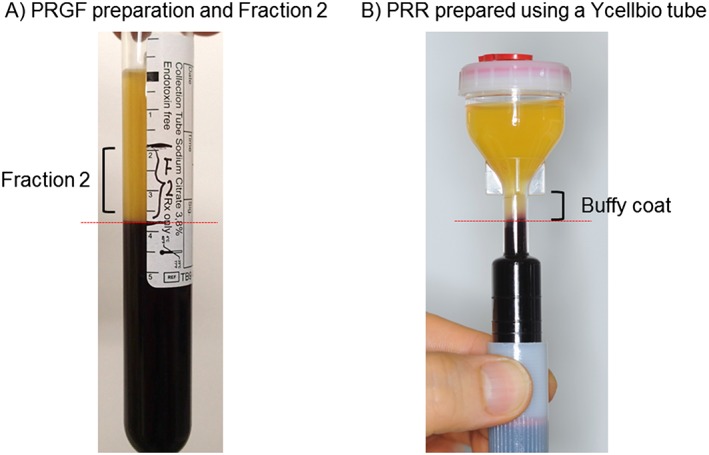
Images of PRGF and PRP preparations produced using a vacuum blood collection tube for PRGF preparation (A) or a Ycellbio PRP preparation tube (B), respectively. PRGF, plasma rich in growth factors; PRP, platelet‐rich plasma.

According to the manufacturer's instructions, blood samples were collected from seven healthy volunteers (male; 37–68 years old) using 18G needles and PRGF‐Endoret® Tubes (BTI Biotechnology Institute, S.L., Miñano, Spain) containing 0.2 mL of sodium citrate. The tubes were centrifuged at 580 *g* for 8 min. Fraction 2 (the fraction above the interface of the red thrombus fraction) was collected as PRGF in this study (Fig. 1A) and subjected to the following experiments.

### Preparation of platelet‐rich plasma

An anticoagulant, acid citrate dextrose (1.5 mL) (ACD‐A; Terumo, Tokyo, Japan), was added to syringes equipped with 18G needles, and blood samples (12.0 mL) were collected from the same volunteers. Because of its high efficiency, PRP was prepared using Food and Administration‐approved Ycellbio PRP preparation tubes (YCELLBIO MEDICAL CO., LTD., Seoul, Korea) (Fig. 1B). Briefly, according to the manufacturer's instructions, freshly collected blood samples were transferred to funnel‐shaped tubes and centrifuged at 1800 *g* for 4 min. After adjusting the level of the buffy coat, the tubes were centrifuged for an additional 4 min. The resulting PRP fractions were collected using syringes equipped with long needles (*N* = 14).

The study design and consent forms for all procedures performed with the study subjects were approved by the ethical committee for human subject use at Niigata University School of Medicine in accordance with the Helsinki Declaration of 1975 as revised in 2008.

### Determination of blood cell counts

The numbers of blood cells in whole blood samples, PRGF preparations, and PRP preparations were determined using an automated hematology analyzer (pocH‐100iV diff; Sysmex, Kobe, Japan). First, red blood cells (RBCs), WBCs, and platelets were counted immediately after blood collection. Second, freshly prepared PRGF and PRP samples were used for the blood cell count. The obtained values were adjusted by their relative dilution factors.

### Evaluation of cell proliferation

Human alveolar bone‐derived periosteal cells were isolated and cultured as described in the succeeding discussion. With informed consent, human periosteum tissue segments were aseptically dissected from the periodontal tissues of the healthy buccal side of the retromolar region of the mandibles of nonsmoking volunteers (Kawase et al. 2009). Small periosteum pieces were expanded to form cell‐multilayered periosteal sheets (*ϕ*30–40 mm) in humidified 5% CO_2_, 95% air at 37°C with Medium 199 (Invitrogen, Carlsbad, CA) supplemented with 10% fetal bovine serum (FBS) (Invitrogen), 25 µg/mL ascorbic acid 2‐phosphate, and antibiotics. Then, the periosteal sheets were enzymatically digested to release single cells. The resulting cells were further expanded in Dulbecco's Modified Eagle's Medium (DMEM) supplemented with 10% FBS.

Single cells were seeded at a density of 1 × 10^4^ in 6‐well plates and pre‐cultured for 24 h in 1% FBS‐containing DMEM. The medium was replaced with the same fresh medium containing PRGF or PRP (0.31–10%), and the cells were cultured for 48 h. Cells were then photographed and counted in four randomly selected views using image‐pro plus software (Media Cybernetics Manufacturing, Warrendale, PA).

All subjects enrolled in this study responded positively to an informed consent that was approved on 22 June 2006, by the Ethics Committee for Human Subject Use at Niigata University Medical and Dental Hospital in accordance with the Helsinki Declaration of 1975 as revised in 2008.

### Statistical analysis

After a normality test, the statistical significance of differences among individual groups was analyzed using Student's *t*‐test for two groups or one‐way analysis of variance for three or more groups as implemented in sigmaplot software (Version 12.5; Systat Software, Inc., San Jose, CA). Comparisons between individual groups were determined using Tukey's multiple comparison test. *P*‐values < 0.05 were considered significant.

Relationships among the three cell components were analyzed by simple linear regression. Linear correlation coefficients were calculated using sigmaplot software. When the *R*‐value was within the 0.7–0.9 and 0.40–0.69 ranges, the correlations were considered strong and moderate, respectively. When *R* was less than 0.4, the correlation was considered weak and not significant.

## Results

**Figure 2:**
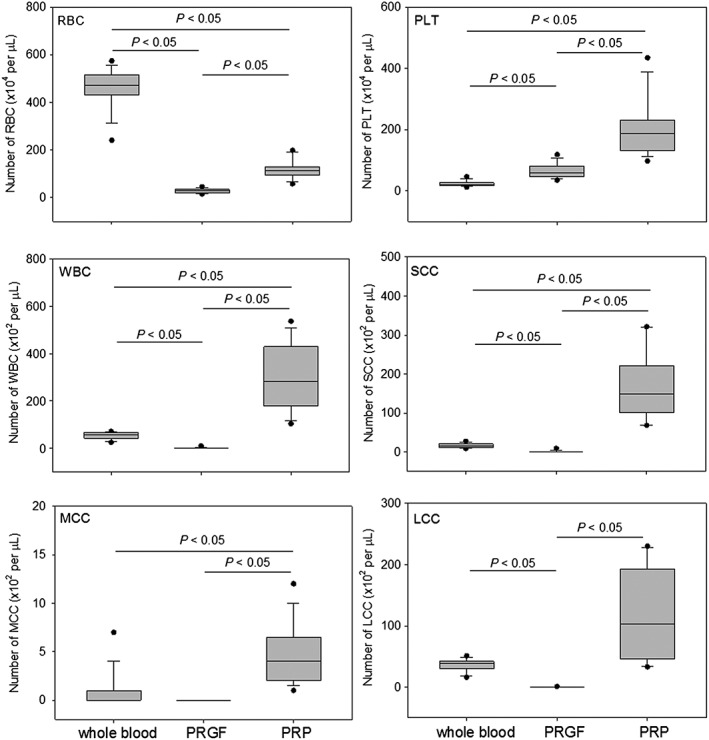
The number of RBCs, WBCs (SCC, MCC, and LCC), and platelets in whole blood samples, PRGF preparations, and PRP preparations. The center lines of the boxes represent medians (*N* = 14). SCC: lymphocytes, MCC: monocytes, and LCC: gametocytes. RBC, red blood cells; WBCs, white blood cells; PRGF, plasma rich in growth factors; SCC, small white blood cells; MCC, middle white blood cells.

Comparisons of RBC, WBC, and platelet counts between PRGF and PRP preparations are shown in Figure 2. In the PRGF preparations, both RBCs and WBCs were almost completely eliminated, and platelets were significantly concentrated by 2.84‐fold. Conversely, in the PRP preparations, the concentration rates of platelets and WBCs in the PRP fractions were 8.79‐ and 5.51‐fold over the whole blood samples, respectively. For the WBC counts, lymphocytes (SCC: small white blood cells) were the most concentrated by 10.2‐fold, whereas monocytes (MCC: middle white blood cells) and gametocytes (LCC: large white blood cells) were 4.27‐ and 3.30‐fold, respectively.

**Figure 3:**
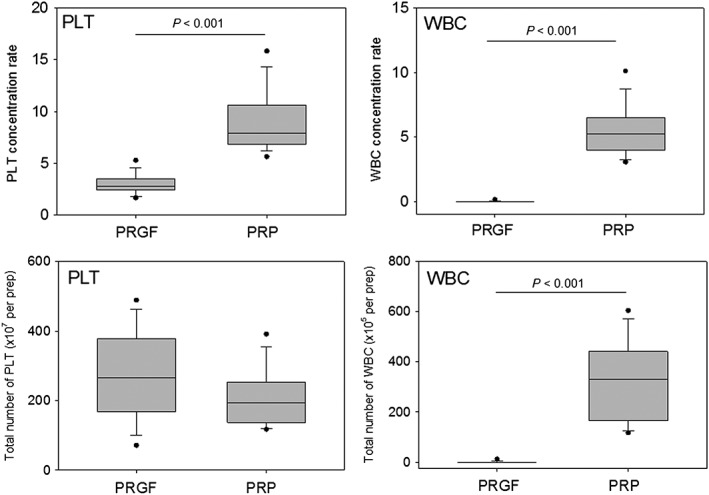
Concentration rates and number of platelets and WBCs in whole blood samples and PRGF fractions. The center lines of the boxes represent medians (*N* = 14). WBC, white blood cell; PRGF, plasma rich in growth factors; PRP, platelet‐rich plasma; PLT, platelet.

The concentration rates of platelets and WBCs and the total numbers of platelets and WBCs per preparation were compared between PRGF and PRP fractions in Figure 3. Although the concentration rate of platelets was substantially higher in the PRP fractions than in the PRGF fractions, the total number of platelets per preparation was slightly higher in the PRGF fractions compared with the PRP fractions. WBCs were concentrated in the PRP fractions, whereas WBCs were excluded from the PRGF fractions.

**Figure 4:**
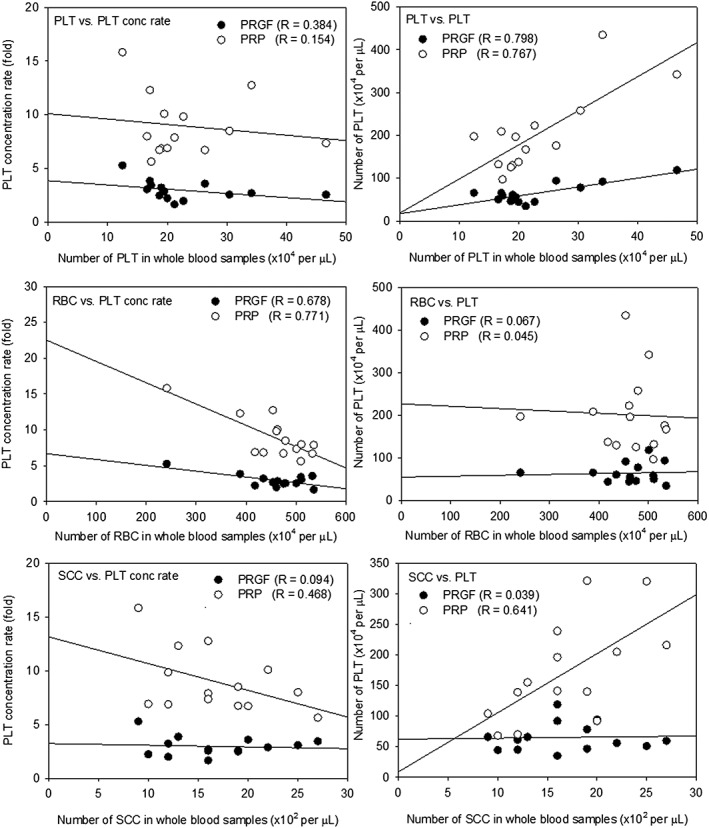
Correlations between blood cell components in whole blood samples and platelet concentration rates or platelet counts in PRGF and PRP preparations (*N* = 14). RBC, red blood cells; PRGF, plasma rich in growth factors; PLT, platelet; PRP, platelet‐rich plasma; SCC, small white blood cells.

The correlations between blood cell components (platelets, RBCs, and lymphocytes) in whole blood samples and platelet counts and concentration rates in the resulting PRGF and PRP preparations are shown in Figure 4. Significantly positive correlations were observed between platelet counts in whole blood samples and those in the PRGF and PRP preparations [Pearson correlation coefficient values (*R*) = 0.798 and 0.767, respectively]. RBC counts in the whole blood samples were negatively correlated with the platelet concentration rates in the PRGF and PRP preparations. In addition, lymphocyte counts in the whole blood samples influenced the platelet concentration rate (*R* = 0.468) negatively and platelet counts (*R* = 0.641) positively only in the PRP preparations.

**Figure 5:**
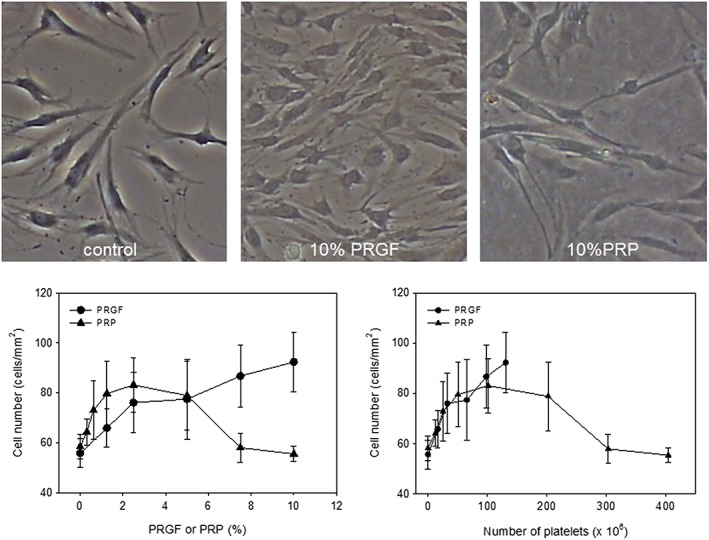
Effects of PRGF and PRP preparations on the proliferation of human periosteal cells. Cells were treated with PRGF or PRP at the indicated doses for 48 h in 1% FBS‐containing medium. (A) Representative images of the cells, (B) dose (%)–response curves for PRGF‐treated or PRP‐treated periosteal cells (*N* = 4), and (C) platelet count–response curves for PRGF‐treated or PRP‐treated periosteal cells (*N* = 4). PRGF, plasma rich in growth factors; PRP, platelet‐rich plasma.

The effects of PRGF and PRP preparations on the proliferation of human periosteal cells are shown in Figure 5. After 48‐h treatments, the PRGF preparations (0.31–10%) increased the number of periosteal cells in a dose‐dependent manner. In contrast, the PRP preparations (0.31–10%) showed a biphasic effect on cell proliferation with maximal effects at 2.5%. However, when the doses (*x*‐axis) were normalized by conversion to platelet counts, the effects of PRGF appeared to be almost identical to those of low doses of PRP. Therefore, to further examine the possible dependency of these phenomena on platelet counts, we performed an additional experiment using platelet‐concentrated PRGF preparations. As shown in Figure S1, even PRGF preparations containing the highest platelet counts, which were equal to 40% of the original PRGF preparations, still stimulated proliferation of the periosteal cells. However, PRP preparations exhibited the biphasic effect that was particularly evident at higher platelet counts.

## Discussion

The specific gravities of RBCs, WBCs, platelets, and plasma are 1.095–1.101, 1.055–1.095, 1.058, and 1.024–1.030, respectively (Malchesky 1996). Based on these values, the theoretical order of the individual fractions from the bottom to the top of the centrifugation tube during PRP preparation is the RBC fraction, the WBC fraction, and the platelet fraction. However, because of the properties of the fractions and the cells, such as the viscosity of the plasma, cell size, and cell deformability, individual blood cell types cannot clearly be fractionated. Therefore, the upper part of the RBC fraction below the buffy coat also contains WBCs and platelets, and the buffy coat contains not only platelets but also WBCs.

**Figure 6:**
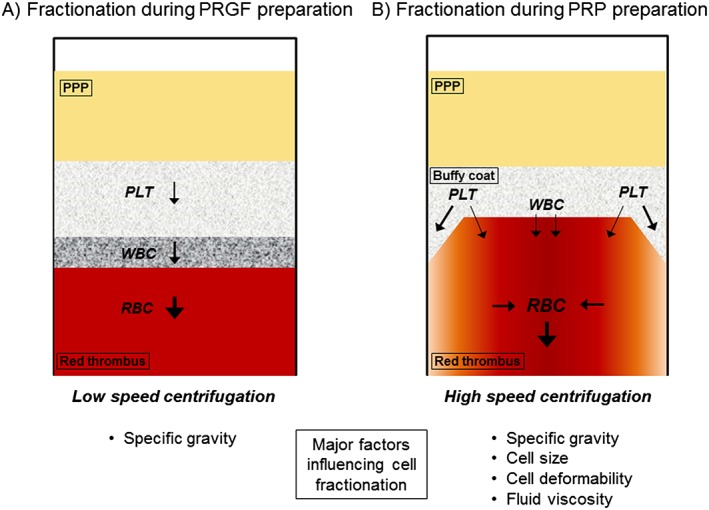
Scheme for wall‐normal force and volume exclusion that may occur in a tube. RBC, red blood cells; PLT, platelet; WBC, white blood cell; PPP, platelet‐poor plasma.

In this study, we demonstrated that relatively slow centrifugation for the preparation of PRGF was not suitable for forming a clear buffy coat, which resulted in low platelet concentration rates, but it was beneficial for separating platelets from WBCs. Therefore, WBC counts in the whole blood samples did not significantly influence platelet fractionation in the PRGF preparations. However, RBC counts in the whole blood samples somehow significantly reduced the platelet concentration rates in both the PRGF and PRP preparations. In blood vessels in which shear flow occurs, RBCs experience a wall‐normal force that arises because of their deformation and propels them away from the wall. By volume exclusion, however, platelets marginate toward the wall (Vahidkhah et al. 2014). During centrifugation of blood samples contained in tubes, similar phenomena may take place depending on the centrifugation speed, and this affects the distribution of platelets and RBCs, especially near the tube walls. This possible mechanism is illustrated in Figure 6. During slow centrifugation, similar to PRGF preparation, it is theoretically thought that blood cells are fractionated mainly by their specific gravities. Therefore, cell fractions that overlap each other are rarely obtained. However, in a case of fast centrifugation, it is thought that blood cells are subject to various influences and that cell fractions can overlap each other.

Recently, studies have proposed that the optimal concentration of platelets for tissue regeneration would be at most approximately threefold higher than that of whole blood (Weibrich et al. 2004; Graziani et al. 2006; Rappl 2011). The over‐concentration of platelets reduces the positive effects of PRP preparations on tissue regeneration. Our data obtained from PRP‐treated cell cultures supported this concept; however, those of PRGF‐treated cell cultures suggested that other components in the PRP preparations, rather than platelets, may interfere with the expected PRP‐induced cell growth.

Based on their belief that WBCs should be eliminated, Anitua and his research group developed PRGF (Anitua et al. 2007). In this study, we demonstrated that the platelet concentration rate in the PRGF preparations was in the optimal range. Even though platelets were concentrated in PRGF preparations, PRGF preparations still maintained their mitogenic action. In addition, even though platelet‐poor plasma was added to diluted original PRP preparations two to three times, we preliminarily observed that higher doses (~10%) of the diluted PRP preparations still significantly reduced the stimulatory effects of PRP observed at lower doses (Kawase et al. unpublished observations). On the other hand, a recent article demonstrated that dopamine and serotine released from activated platelets are necessary to endogenous stem cell recruitment and subsequent dentin repair (Baudry et al. 2015). These monoamines may suppress the proliferation of target cells while accelerating cell differentiation. Therefore, at present, we speculate that a factor(s) contained in PRP preparations, but not in PRGF preparations, such as WBC‐derived factors, is the most involved in the reduction of the accelerated proliferation. However, it cannot be ruled out that other unidentified components released from activated platelets may also solely or cooperatively influence the periosteal cell proliferation. Further investigation should be performed to identify the key factors involved in this phenomenon.

## Conclusions

The inter‐individual efficacy and potency of PRP preparations has been explained solely by the individual‐dependent levels of growth factors. In this study, the statistical analysis demonstrated that WBCs and platelets could be concentrated similarly in the individual PRP preparations. However, we also found that slow centrifugation can remove WBCs from the platelet fraction during PRGF preparation. Because WBCs have often been indicated as a negative potent factor for tissue regeneration, the individual‐dependent differences in WBC counts could be an alternative or additional explanation for the inter‐individual efficacy differences in PRP preparations. PRGF preparation, in which platelets are optimally concentrated and WBCs are excluded, may be a more appropriate application for tissue regeneration than PRP preparations.

## Conflicts of Interest

The authors declare that they have no competing interests.

## Supporting information


**Figure S1.** Effects of platelet‐concentrated PRGF preparations on the proliferation of human periosteal cells. Blood samples were collected from healthy male volunteers (age: 26 and 48‐years old) and centrifuged to prepare PRGF fraction 2. This fraction was further centrifuged to concentrate platelets by 4‐fold. The number of platelets were counted at the end of each step. These platelet‐concentrated PRGF preparations or the normal PRP preparations were added to cell culture medium at concentrations of 1.25%, 2.5%, 5% or 10% (w/v) and cell numbers were evaluated by image analysis. PPP preparations were added as control at a dose of 5% or 10%. Differences between two groups were assessed by Student's t‐test. When normality testing failed, a Mann‐Whitney Rank Sum test was performed. A *p*‐value of less than 0.05 was considered to be statistically significant. N = 4 – 7.

Supporting info item
