# Supplementary material for: Basic characteristics of plasma rich in growth factors (PRGF): blood cell components and biological effects
Source: Clin Exp Dent Res. 2016 Mar 18;2(2):96–103. doi: 10.1002/cre2.26 (PMC5839250; doi:10.1002/cre2.26)

***Supplemental data***

**Figure S1.** Effects of platelet-concentrated PRGF preparations on the proliferation of human periosteal cells. Blood samples were collected from healthy male volunteers (age: 26 and 48-years old) and centrifuged to prepare PRGF fraction 2. This fraction was further centrifuged to concentrate platelets by 4-fold. The number of platelets were counted at the end of each step. These platelet-concentrated PRGF preparations or the normal PRP preparations were added to cell culture medium at concentrations of 1.25%, 2.5%, 5% or 10% (w/v) and cell numbers were evaluated by image analysis. PPP preparations were added as control at a dose of 5% or 10%. Differences between two groups were assessed by Student’s t-test. When normality testing failed, a Mann-Whitney Rank Sum test was performed. A p-value of less than 0.05 was considered to be statistically significant. N = 4 – 7.


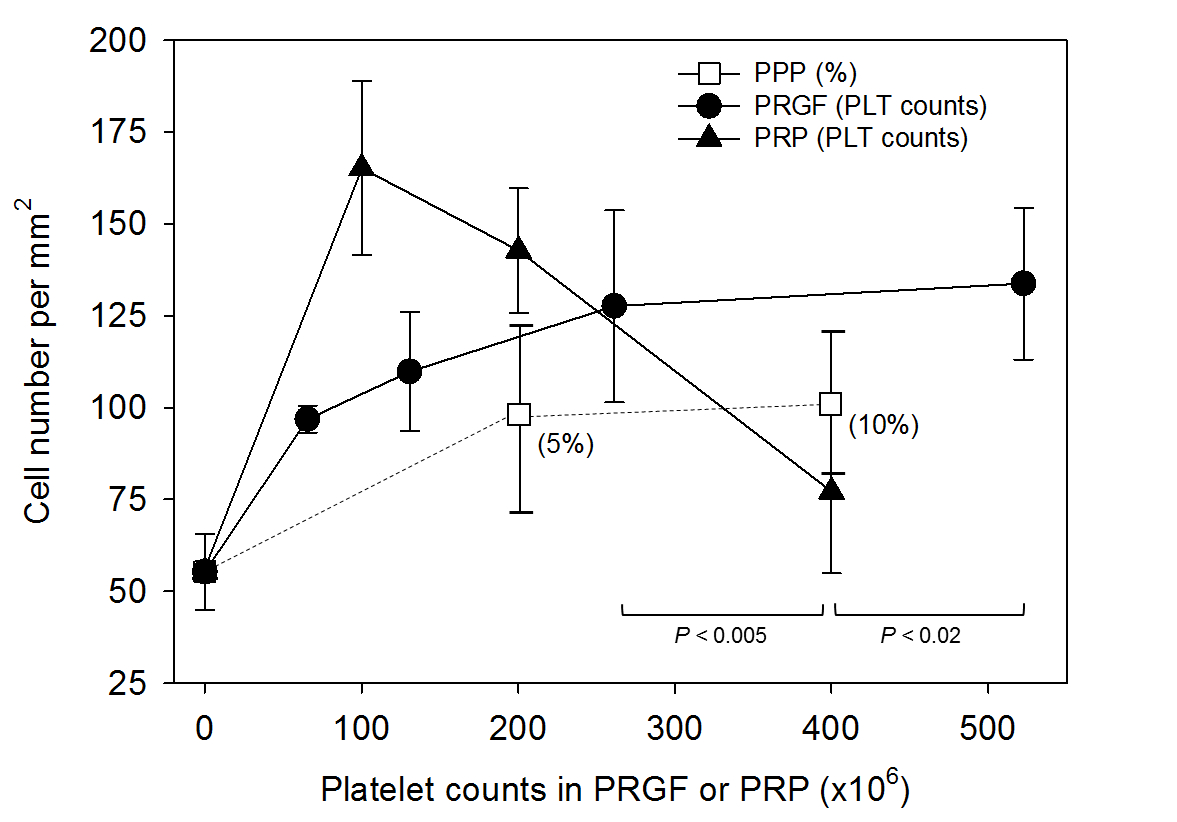

Supplement: Supplementary file 1 — Supporting info item [file CRE2-2-96-s001.docx]
